# Surface Engineering of Triboelectric Nanogenerator with an Electrodeposited Gold Nanoflower Structure

**DOI:** 10.1038/srep13866

**Published:** 2015-09-14

**Authors:** Sang-Jae Park, Myeong-Lok Seol, Seung-Bae Jeon, Daewon Kim, Dongil Lee, Yang-Kyu Choi

**Affiliations:** 1 Department of Electrical Engineering, KAIST, 291 Daehak-ro, Yuseong-gu, Daejeon 305-701, Republic of Korea

## Abstract

A triboelectric nanogenerator composed of gold nanoflowers is demonstrated. The proposed triboelectric nanogenerator creates electricity by contact-separation-based electrification between an anodic metal and a cathodic polymer. For the improvement of output power via the enlargement of the effective surface area in the anodic metal, gold nanoflowers that produce a hierarchical morphology at a micro-to-nano scale by electrodeposition are utilized. The hierarchical morphology is controlled by the applied voltage and deposition time. Even though the triboelectric coefficient of gold is inferior to those of other metals, gold is very attractive to make a flower-like structure by electrodeposition. Moreover, gold is stable against oxidation by oxygen in air. From a reliability and practicality point of view, the aforementioned stability against oxidation is preferred.

Recently, to supply sustained power for electronic devices, energy harvesting has drawn considerable attention. Energy harvesting converts waste energy to electrical energy. There are various types of waste energy sources, such as thermal, light, and mechanical energy. In particular, mechanical energy has attracted great interest because of its abundance in nature. To convert waste mechanical energy to useful electrical energy, various methods have been introduced, such as an electromagnetic^1^,^2^,^3^,^4^ electrostatic^5^,^6^,^7^,^8^, and piezoelectric^9^,^10^,^11^,^12^ methods. Alternatively, a novel, eco-friendly, highly reliable triboelectric nanogenerator (TENG) fabricated with low cost is under active development^13^.

The key mechanism of the TENG is the contact electrification that occurs at the interface between two different materials, such as polymer and metal. To enhance the resultant surface charge density of the contact electrification process, the effective contact surface area should be enlarged by surface engineering with micro and nanostructure. In particular, many studies have introduced polymer surface engineering with micro- and nanostructures using various processes, such as template-based molding and plasma treatment^14^,^15^,^16^,^17^,^18^,^19^,^20^,^21^,^22^,^23^. On the other hand, there have been few related studies on metal surfaces because of limited fabrication methods.

Gold is used in various applications, such as bio- or chemical-sensors, and it is highly robust against oxygen in air, while most metals are easily corroded and oxidized. In particular, surface modification of gold is more readily achieved by electrodeposition in comparison with other metals. Gold electrodeposition easily makes a large surface area with a flower-like structure under room temperature and atmospheric pressure without complex fabrication processes. Historically, gold nanoflowers obtained by electrodeposition have mostly been used to enhance sensor performance in surface-enhanced Raman spectroscopy (SERS)^24^,^25^ and microelectrode arrays (MEA)^26^,^27^; however, they have never been used to improve the output power of TENGs. Moreover, the shape, surface roughness, and electrochemical characteristics of gold nano-structures can be controlled by the applied voltage, electrodeposition time, and solution concentration^28^,^29^. Also, it is advantageous to employ gold nanoflowers in TENGs because no special protection package is required in an air environment due to gold’s robustness against oxygen. It should be noted that protective passivation is mandatory in sensor applications; however, this is not the case in TENGs because TENGs harvest waste energy from various environments. Thus, the inherent surface stability of gold is preferred in TENG applications.

In this paper, using the gold electrodeposition method, a surface engineered gold nanoflower triboelectric nanogenerator (simply AuNF TENG) is introduced. With variation of the applied voltage and deposition time, the AuNF TENG shows different surface shapes and varying performance. The surface engineered AuNF TENG used as an experimental sample shows 1.5 to 2 times higher open-circuit voltage and short-circuit current compared with an Au flat TENG without the micro- and nano-morphology, which is used as a control sample. Moreover, the AuNF electrode produces higher output power than flat aluminum and copper electrodes despite the inferior triboelectric coefficient of gold. The AuNF TENG shows high robustness against hot and humid environments. It also shows good endurance after iterative cyclic operations.

## Results

### Device structure

**Figure 1:**
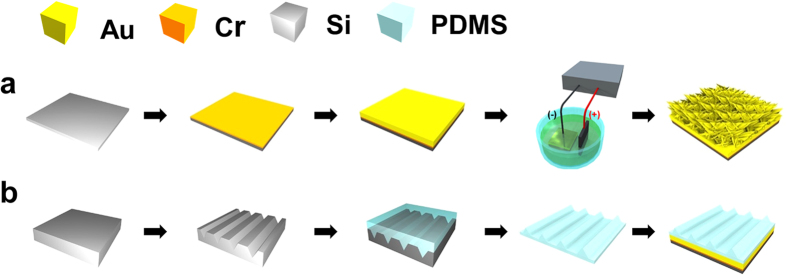
Schematic of AuNF TENG fabrication. (**a**) Fabrication process of the bottom part. 100 nm Cr and 150 nm Au are deposited on Si wafer by sputtering. AuNF structures are formed on the deposited Au layer. (**b**) Fabrication process of the top part. Triangular line grooves are formed in Si wafer by KOH etching. PDMS is subsequently filled, hardened, and detached. Then, the resultant PDMS layer with triangular line is attached to the electrode.

The AuNF TENG consists of two electrodes; one is a bottom electrode which contains the AuNF, and the other is a top electrode which contains triangular-line-shaped polydimethylsiloxane (PDMS). The AuNF surface was controlled by variation of the applied voltage and electrodeposition time (Fig. 1a). Triangular-line-shaped PDMS was fabricated by using silicon lithography and etching process (Fig. 1b). The size of the AuNF TENG was 2 cm × 1.8 cm.

### Working principle

**Figure 2:**
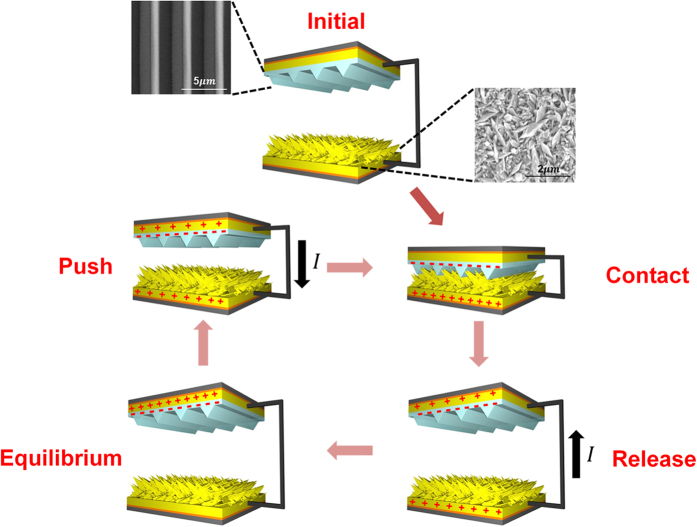
Operation mechanism of the AuNF TENG. In the initial state, the AuNF and the triangular-line-shaped PDMS are separated from each other. External force causes the AuNF and triangular-line-shaped PDMS into contact. The AuNF is charged positively, and the triangular-line-shaped PDMS is charged negatively by triboelectric characteristics. Removal of external force causes separation, and electrons flow from the triangular-line-shaped PDMS to the AuNF electrode. When the AuNF and the triangular-line-shaped PDMS are separated by a greater distance, electrical equilibrium is formed. External force is applied again to make the AuNF and the triangular-line-shaped PDMS come into contact; thus, it induces electrons to flow from the AuNF electrode through external load to the triangular-line-shaped PDMS.

The aforementioned contact and separation method was applied to characterize the AuNF TENG (Fig. 2). In the initial state, the top electrode and the bottom electrode are separated from each other; hence, there is no charge distribution. When two electrodes come closer and contact each other, charge redistribution occurs between the AuNF and the triangular-line-shaped PDMS. As a consequence, the triangular-line-shaped PDMS is charged negatively, whereas the AuNF is charged positively owing to its intrinsically different triboelectricity tendency. In separation mode, two electrodes are separated, and current flows from the bottom electrode to the top electrode for a new equilibrium state. In contact mode again, two electrodes are approaching closer, current flows from the top electrode to the bottom electrode for another new equilibrium state. By these iterative modes of contact and separation, alternating current (AC) is produced.

### Electric measurement

**Figure 3:**
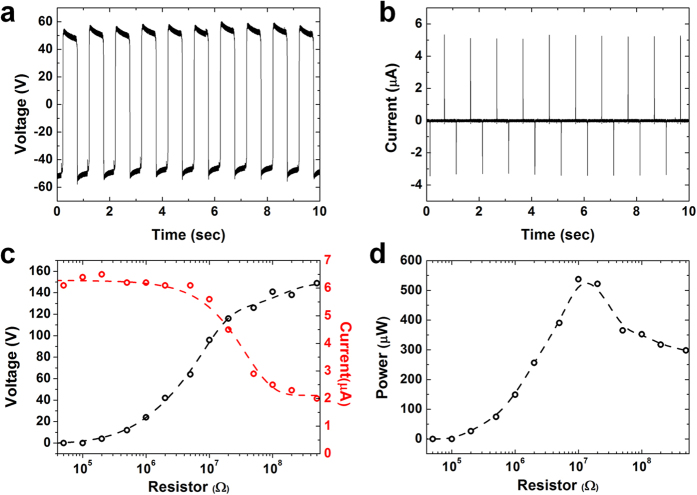
Electrical performance of the AuNF TENG. (**a**) Open-circuit voltage of the AuNF TENG. (**b**) Short-circuit current of the AuNF TENG. (**c**) Open-circuit voltage and short-circuit current of the AuNF TENG with various load resistances. (**d**) Power of the AuNF TENG with various load resistances. The maximum power of 540 μW (i.e., 150 μW/cm^2^) was obtained with 10 MΩ load resistance.

The typical AuNF TENG performance was measured (Fig. 3). The maximum open-circuit voltage was approximately 110 V (Fig. 3a), while the maximum short-circuit current was approximately 5.5 μA (Fig. 3b). To calculate the maximum power of the AuNF TENG, the open-circuit voltage and short-circuit current were measured for various load resistances (Fig. 3c). When the load resistance was 10 MΩ, the maximum power of 540 μW (i.e., 150 μW/cm^2^) was obtained (Fig. 3d). The maximum power from the AuNF TENG is able to turn on more than 70 light emitting diodes (LEDs) directly without any rectifying circuit or storage capacitor.

### Surface engineering

**Figure 4:**
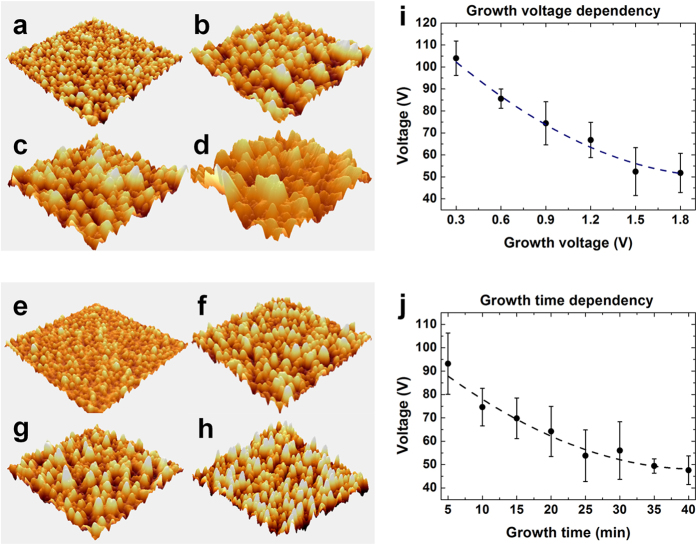
Analyses of growth parameter dependence. AFM images with various growth voltages: (**a**) 0.3 V, (**b**) 0.9 V, (**c**) 1.5 V, and (**d**) 1.8 V. AFM images with various growth times: (**e**) 5 min, (**f**) 15 min, (**g**) 25 min, and (**h**) 35 min. (**i**) Output open-circuit voltage with AuNF TENGs with various growth voltages. (**j**) Output open-circuit voltage with AuNF TENGs with various growth times.

The output performance will be affected by various AuNF surfaces. From flat gold surface to a certain optimal AuNF surface, output performance will be increased by increment of the effective contact area. Beyond the optimal point to grow the AuNF structure, however, output performance will be decreased because too rough AuNF surface makes a partial tip-to-tip contact between the AuNF and the triangular-line-shaped PDMS, i.e., incomplete contact. There are three factors that can be varied to modify the AuNF surface. The first factor is the applied DC voltage, the second factor is the electrodeposition time, and the third factor is the relative concentrations of HAuCl_4_, PVP, and DI water in the mixed solution. The applied voltage and the electrodeposition time do not seriously deform the geometric shapes of the gold nanoflowers, while the solution concentrations distort the overall shapes of the gold nanoflowers. For this reason, the applied voltage and electrodeposition time dependency were investigated with a fixed HAuCl_4_ concentration of 15 mM to determine how morphology changes can influence triboelectricity. Firstly, the growth voltage dependency was investigated. Different AuNF TENGs were fabricated by variation of the applied voltage from 0.3 V to 1.8 V with 0.3 V intervals and the fixed electrodeposition time of 25 min. Higher applied voltages resulted in greater roughness. However, when the micro-structure became too tall, the output voltage was degraded (Fig. 4i). In growth voltage dependency, 0.3 V (in fixed 25 min) condition was the optimal point. Secondly, the growth time dependency was analyzed. Dissimilar AuNF TENGs were fabricated by varying the growth time from 5 min to 40 min with 5 min intervals and the fixed applied voltage of 0.9 V. Similarly, longer electrodeposition times resulted in greater roughness. However, when the micro-structure became too tall, the output voltage decreased (Fig. 4j). In growth time dependency, 5 min (in fixed 0.9 V) condition was the optimal point. The detailed TENG performance results in relation to growth voltage and growth time. They are provided in the supporting information (Figure S1). There is a reciprocal relationship in the output voltage versus the height difference between the maximum height and minimum height of the nanoflower as shown in Figure S2. Similarly, there is a reciprocal relationship in the output voltage versus the root mean square roughness (R_q_) as shown in Figure S2. As expected, the effective surface area expanded as the surface roughness increased. In general, a larger contact area produced higher triboelectricity. However, too much roughness induced tip-to-tip contact between the AuNF and the triangular-line-shaped PDMS; hence, this caused incomplete contact. Therefore, the triboelectricity was unintentionally decreased. When the growth voltage was 1.8 V and the growth time was 40 min, the magnitude of the triboelectricity was even lower than that of the Au plate TENG (control group). Thus, optimal surface engineering is necessary for the maximization of triboelectricity. In this surface engineering, the electrodeposition voltage of 0.3 V for 25 min and electrodeposition time 5 min for 0.9 V show the optimal surface in each growth dependency.

### Comparison with other metals

**Figure 5:**
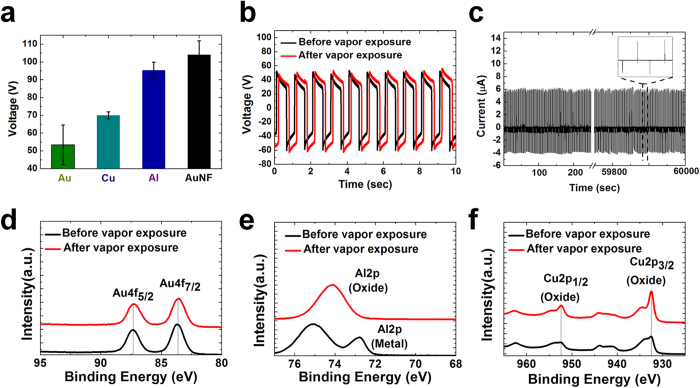
(**a**) Output open-circuit voltages of TENGs with flat Au, flat Cu, flat Al, and AuNF at the contact interface. The AuNF TENG produced the best output performance. (**b**) Output open-circuit voltage of the AuNF TENG before and after vapor exposure. Oxidation robustness of AuNF can be confirmed. (**c**) Short-circuit current of the AuNF TENG over 60,000 contact separation cycles (60,000 sec). XPS data of (**d**) AuNF (**e**), Al, and (**f**) Cu TENGs before and after vapor exposure. Excellent oxidation robustness of Au is confirmed.

As mentioned earlier, gold tends to be less positively charged for TENG application in comparison with other metals. To overcome this weakness, the optimal AuNF TENG was made by gold electrodeposition. The optimal AuNF TENG showed better TENG performance than both the flat gold electrode-based TENG and the flat aluminum and copper electrode-based TENGs (Fig. 5a). On the other hand, it is well known that gold is a noble metal; therefore, it has better resistance to harsh environments than many other metals. To confirm this resistance, an oxidation experiment was conducted. The flat copper TENG, aluminum TENG, and AuNF TENG were exposed to air with 90% humidity at 50 °C in an enclosed bath for 7 hours. After oxidation, the exposed samples were analyzed by X-ray photoelectron spectroscopy (XPS). While oxygen and hydrogen were detected in the flat copper and aluminum TENGs, nothing except Au was found in the AuNF TENG. This means that the flat copper and aluminum TENGs had been oxidized; however, the AuNF TENG had not been oxidized (Fig. 5d–f). Consistently, the performance of the AuNF TENG barely changed, even under harsh conditions of high temperature and humidity (Fig. 5b). In the endurance test, over sixty-thousand cycles of contact and separation, the AuNF TENG showed a nearly constant level of short-circuit current (Fig. 5c). Therefore, the AuNF TENG can be practically utilized as an energy harvester for outdoor use without a particular protection package with long-term reliability.

### Gold thin paper

To further investigate the possibility for versatile applications of the AuNF TENG with very low cost and a flexible Au-coated thin paper, which was commercialized as a decoration or wrapping paper, it was utilized for electrodeposition. As long as gold is coated on a non-specified surface, gold electrodeposition can be possible anywhere. For this experiment, a highly purified gold (>24 k) foiled paper with an area of 4.5 cm × 4.5 cm and a thickness of 100 nm was attached to a flat PDMS substrate. Then, the surface was modified by gold electrodeposition with the growth voltage of 2.0 V, the growth time of 60 min, and the same HAuCl_4_ concentration of 15 mM. A digital image and an SEM image of mushroom-like structures are provided in the supporting information (Figure S3). From this, it can be expected that the proposed technique can contribute to making a large, flexible TENG with extremely low cost.

## Discussion

A gold nanoflower triboelectric nanogenerator (AuNF TENG) fabricated by electrodeposition was demonstrated. When a nanostructure was made with too much roughness, the triboelectricity of the AuNF TENG was degraded because of the decreased contact area related to the tip-to-tip contact between the AuNF surface and the triangular-line-shaped PDMS. To find the optimal AuNF surface morphology, the supplied DC power voltage and electrodeposition time were varied. The optimal surface was made under the electrodeposition voltage of 0.3 V for 25 min and that of 0.9 V for 5 min. The optimal AuNF TENG showed 1.5 to 2 times better performance than the flat gold electrode-based TENG. Moreover, the optimal AuNF TENG showed better TENG performance than the flat copper and aluminum electrode-based TENGs. Moreover, the AuNF TENG, even with a rough profile and enlarged effective surface area, showed better robustness against oxygen in air at high humidity in comparison with the copper- and aluminum-based TENGs, which did not have the micro-to-nano morphology. In particular, this stability is attractive when the TENG is used outdoors.

## Methods

### Fabrication of the AuNF TENG

To fabricate the AuNF bottom electrode, Cr (100 nm) and Au (150 nm) layers were sequentially deposited on a Si wafer by sputtering. Cr improves adhesion between the Au layer and the Si wafer. The sputtered Au layer serves as a seed layer during electrodeposition. Next, the Au/Cr deposited Si wafer and the reference electrode graphite were immersed in a mixed solution of HAuCl_4_ (HAuCl_4_ ·3H_2_O, Sigma Aldrich), 20 g·L^−1^ poly-vinylpyrrolidone (PVP, K 30, Fluka), and deionized (DI) water for electrodeposition. Using a DC power supply (Agilent, E3631A), a positive electrode was wired to the graphite electrode, and a negative electrode was wired to the Si wafer. As a consequence, the AuNF was ready to be used for the bottom electrode. In turn, to fabricate the triangular-line-shaped PDMS, a straight line with a width of 2 μm was delineated by photolithography with photoresist (HKT501) on another bare Si wafer, which was used as a mother template. Then, a triangular line groove was formed on the mother Si wafer by KOH etching. KOH etches silicon with a single crystalline orientation of (111), so the etching process automatically stopped after the trench-type pyramid array was fabricated. To increase the adhesion between the subsequently introduced PDMS and the mother Si template, O_2_ plasma and trichlorosilane were utilized as a surface treatment. Then, the PDMS was filled, hardened, and detached. Finally, the detached triangular line shaped PDMS with the width of 2 μm was ready to be used for the top electrode.

### Characterization

For the characterization of the fabricated AuNF TENG, the contact and separation method was used. One cyclic period of contact and separation was set to 1 sec (contact = 0.5 s, separation = 0.5 s). The applied force from the mechanical shaker was fixed at 100 N. The output voltage and current were measured by an electrometer (Keithley 6514). The load resistance of the electrometer was 200 TΩ for open-circuit voltage measurement and 2 Ω for short-circuit current measurement, respectively.

## Additional Information

**How to cite this article**: Park, S.-J. *et al.* Surface Engineering of Triboelectric Nanogenerator with an Electrodeposited Gold Nanoflower Structure. *Sci. Rep.* **5**, 13866; doi: 10.1038/srep13866 (2015).

## Supplementary Material

Supplementary Information
