# Supplementary material for: Surface Engineering of Triboelectric Nanogenerator with an Electrodeposited Gold Nanoflower Structure
Source: Sci Rep. 2015 Sep 14;5:13866. doi: 10.1038/srep13866 (PMC4568466; doi:10.1038/srep13866)
Supplement: Supplementary Information [file srep13866-s1.doc]

**Supporting Information**

**Surface Engineering of Triboelectric Nanogenerator with an Electrodeposited Gold Nanoflower Structure**

Sang-Jae Park, Myeong-Lok Seol, Seung-Bae Jeon, Daewon Kim, Dong-Il Lee , and Yang-Kyu Choi*

Department of Electrical Engineering, KAIST, 291 Daehak-ro, Yuseong-gu, Daejeon 305-701, Republic of Korea

* Address correspondence to ykchoi@ee.kaist.ac.kr.

**Performance of the AuNF TENG**

Open-circuit voltage and short-circuit current with the AuNF TENGs with various process parameters are shown in Figure S1.


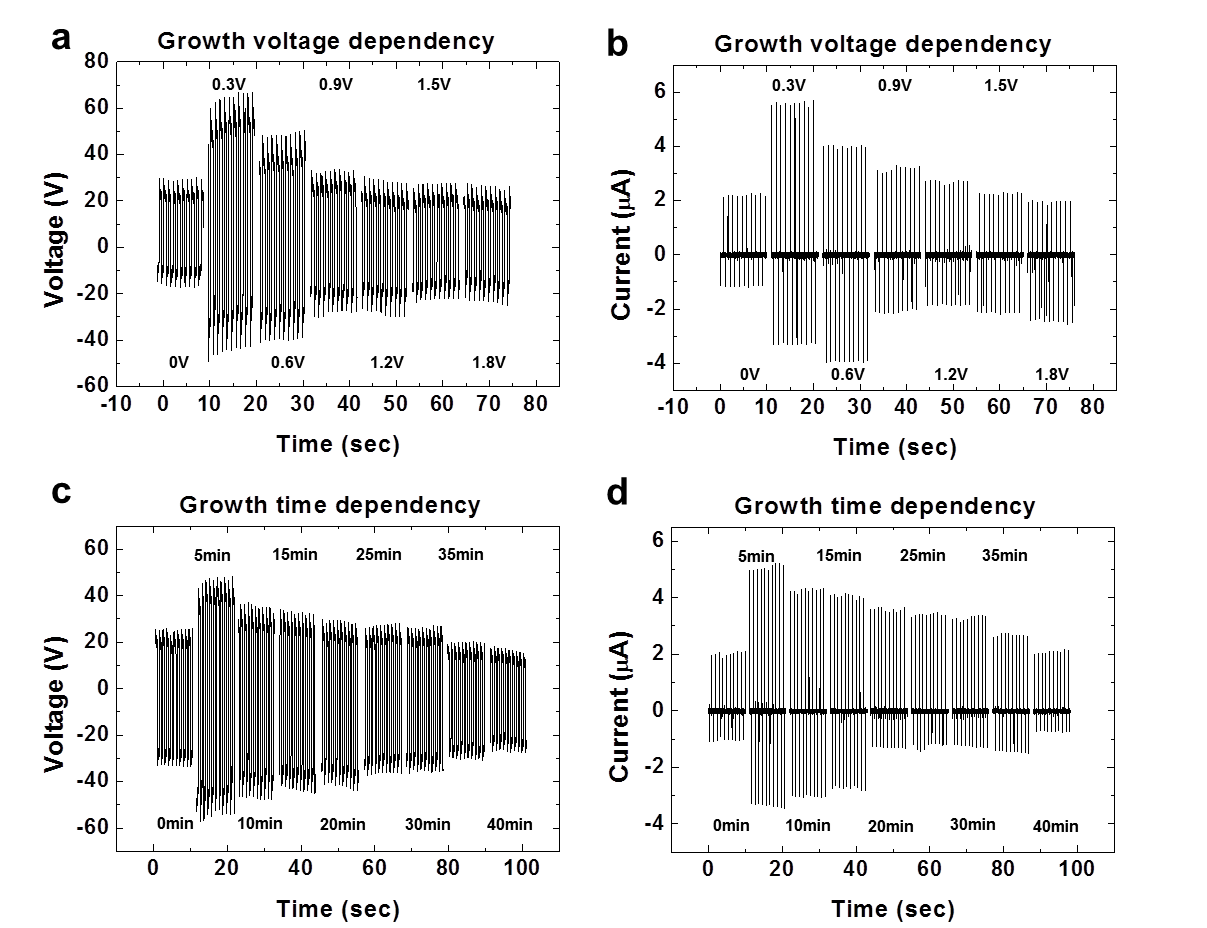


Figure S1. Output open-circuit voltage and short-circuit current spectra of AuNF TENGs with various growth parameters.

The performance results of the AuNF TENGs produced with various growth times and voltages are plotted. The AuNF TENG electrodeposited at 0 V is a flat gold plate without morphology. The AuNF TENG electrodeposited at 0.3 V produced the highest TENG performance. Increased growth voltage degraded the open-circuit voltage and the short-circuit current of the AuNF TENGs (Figure S1a, Figure S1b). Similarly, the AuNF TENG electrodeposited with 5 min of growth time produced the highest TENG performance. Prolonged growth time degraded the open-circuit voltage and short-circuit current of the AuNF TENGs (Figure S1c, Figure S1d).

**Open-circuit voltage in relation to AuNF TENG roughness**

Using AFM (XE-100, Park Systems) analysis, the AuNF surface characteristics were correlated with the output open-circuit voltage and conceptual figure of the incomplete contact between AuNF and triangular-line-shaped PDMS are as shown in Figure S2.


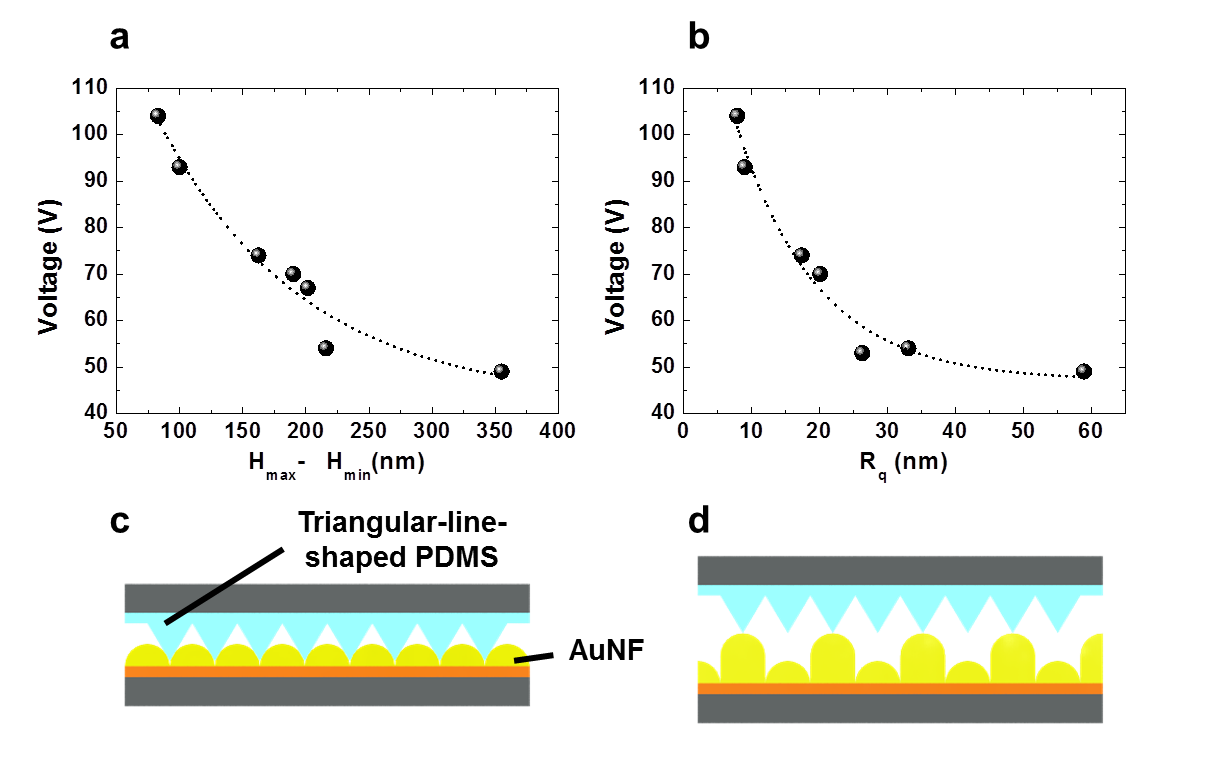


Figure S2. Open-circuit voltage in relation to surface roughness of the AuNF TENG & Conceptual figure of the incomplete contact

Output voltage decreased as the “height difference” increased (Figure S2a). Similarly, the output open-circuit voltage decreases as the root-mean-square roughness increased (Figure S2b). Micro-structures that are too tall with a large aspect ratio are undesirable for TENG performance because only the tips of the structures come into contact. In general, a larger surface area at the contact interface induces larger triboelectric charge density, which results in stronger output power. But, the surface area is not the only parameter to determine output power. An aspect ratio of the protruded micro-to-nano structure should also be considered. If the fabricated AuNF is well fitted to the polymer surface, the entire surface area will be in contact and TENG performances will be improved (Figure S2c). When the aspect ratio of the structure is too large, however, undesired tip-to-tip contact will be made between the AuNF structure and the triangular-line-shaped PDMS (Figure S2d). In this case, only the partial area will be charged, so power enhancement ratio will be degraded. As a result, it can be concluded that TENG performance is closely related to surface roughness.

**Gold thin paper electrodeposition**

To widen the application area of the AuNF TENG, a flexible Au-coated thin paper was utilized for electrodeposition. Digital and SEM images of the utilized sample are shown in Figure S3.


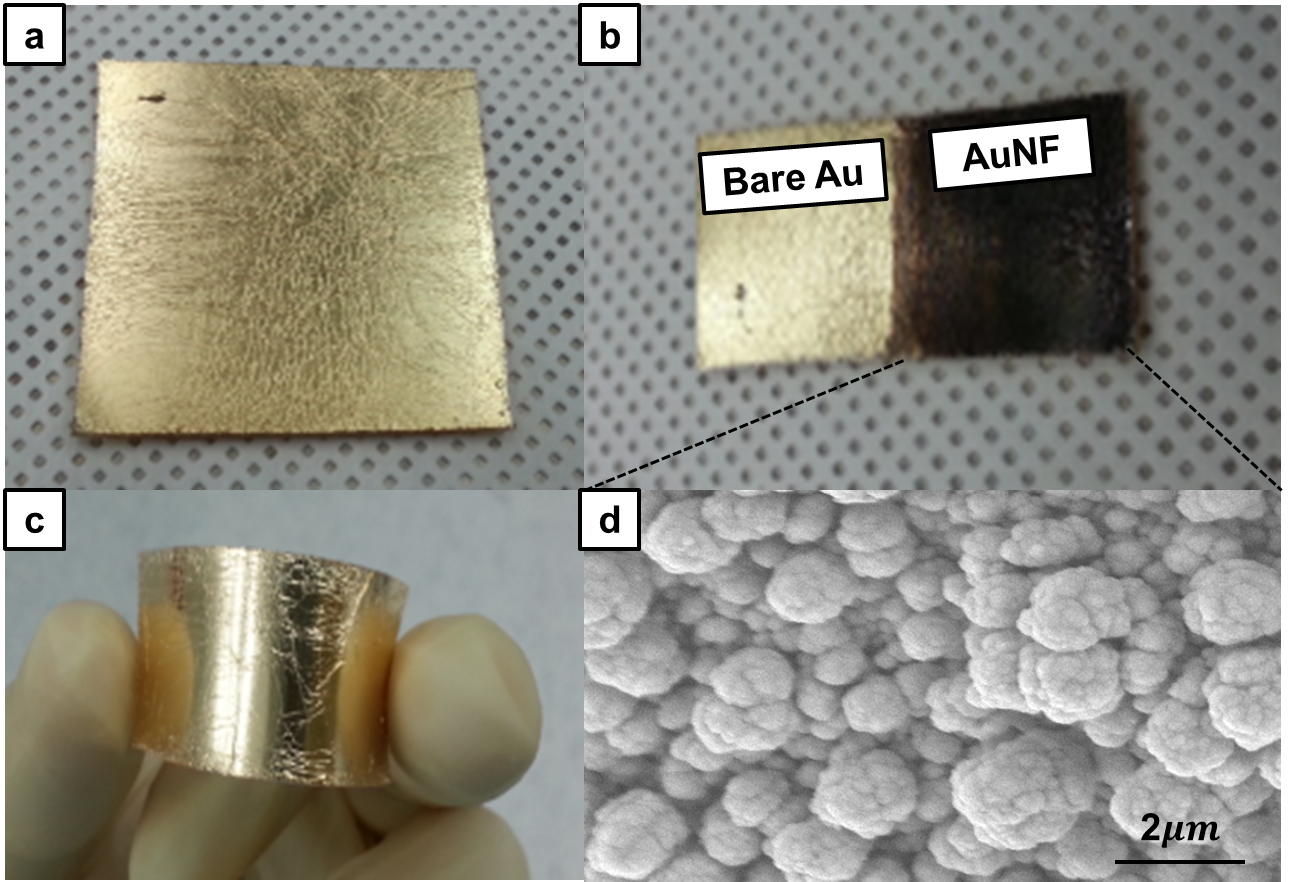


Figure S3. Surface morphology images of the gold thin paper used for electrodeposition

A commercially available gold thin paper (4.5 cm ×4.5 cm×0.00001 cm, 24k) was attached to polydimethylsiloxane (PDMS) (Figure S3a). The Au-coated PDMS showed high flexibility with stable mechanical property (Figure S3b). The Au-coated PDMS was electrodeposited partially with 2.0 V for 60 min at a 15 mM concentration (Figure S3c). High voltage and long electrodeposition time produced a mushroom-like structure without use of any pre-surface treatment or pre-cleaning prior to the electrodeposition (Figure S3d). Under proper conditions, a flexible, large-scale AuNF TENG can be simply utilized with this approach.
